# Diffuse large B cell lymphoma within an anal fistula: a case report with an intriguing possible aetiology

**DOI:** 10.1093/jscr/rjab360

**Published:** 2021-08-16

**Authors:** James Kent, David Joske, Jeremy Parry

**Affiliations:** Bentley Health Service, Department of Surgery, Perth, WA 6102, Australia; Sir Charles Gairdner Hospital, Department of Haematology, Nedlands, WA 6009, Australia; Murdoch University, Department of Pathology, Murdoch, WA 6150, Australia

## Abstract

Described is a case of diffuse large B cell lymphoma that presented within a typical fistula tract, possibly secondary to oxidative stress within the fistula tract itself and consequent malignant change rather than a fistula as a consequence of necrosis in a lymphoma. If so it would be unique in the world literature. A 42-year-old fitness instructor presented with a typical appearing left lateral anal fistula. Biopsy of the fistulous tract revealed B cell lymphoma, graded 1E. Although chemotherapy cured the lymphoma, surgical treatment by ligation of the inter-sphincteric fistula tract was required to heal the fistula. At 3-year follow-up, there has been no recurrence of the lymphoma or the fistula. Neoplasia arising secondary to oxidative stress within an anal fistula is a well-established phenomenon. Early diagnosis of rare conditions associated with anal fistula can only be accomplished by routine biopsy of every fistula tract.

## INTRODUCTION

About 10% of patients with non-Hodgkin’s lymphoma (NHL) will have primary involvement of the gastrointestinal tract [1]. Perirectal sepsis is also a not uncommon presentation of patients with a variety of haematological malignancies where neutropenia supervenes during chemotherapy treatment [2]. In contrast, perianal lymphoma is quite rare [3] with the majority of cases presenting in HIV-positive patients [4]. The first case of anal lymphoma was reported in 1985 by Steele *et al.* [5] with more recent reports of B cell lymphomas of the anal canal in non-immunocompromised patients [6–8]. We report a case of anal lymphoma with the clinical course suggestive of lymphomatous transformation within a pre-existing anal fistula rather than fistula formation of a primary anal lymphoma.

## CASE REPORT

**Figure 1:**
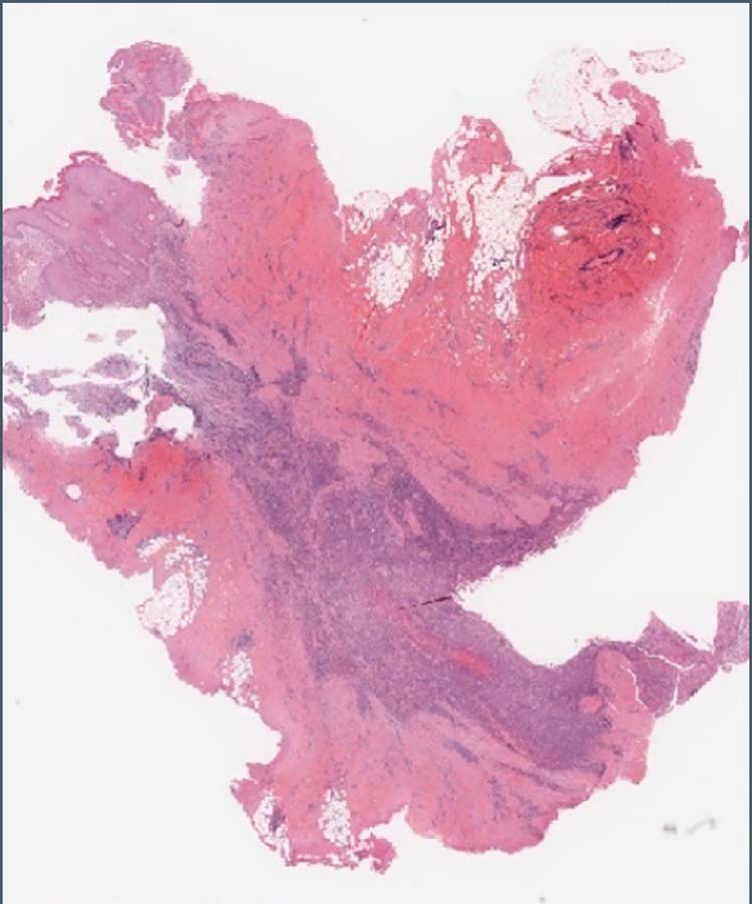
Histology: low-power view showing squamous mucosa (upper left) within an underlying fistula tract associated with a dense cellular infiltrate.

**Figure 2:**
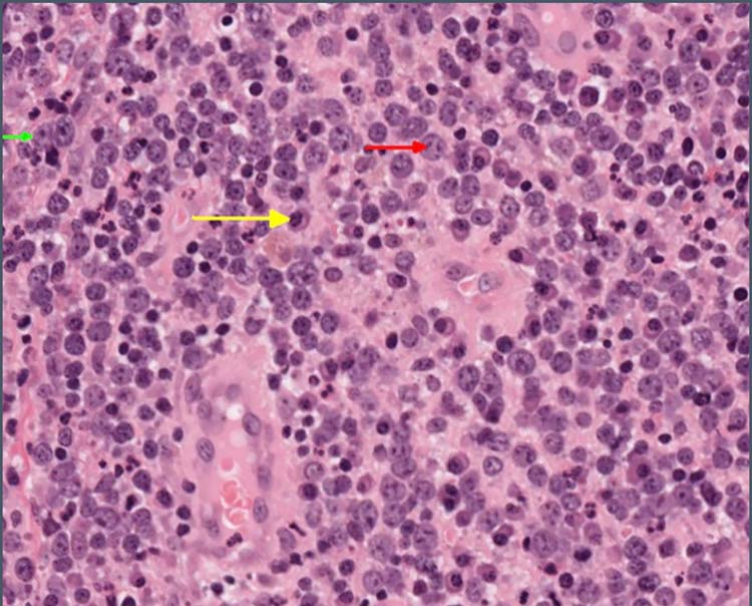
High-power view showing abundant large lymphocytes with the morphology of immunoblasts (small green arrow) and centroblasts (medium red arrow), with reactive plasma cells in the background (long yellow arrow).

**Figure 3:**
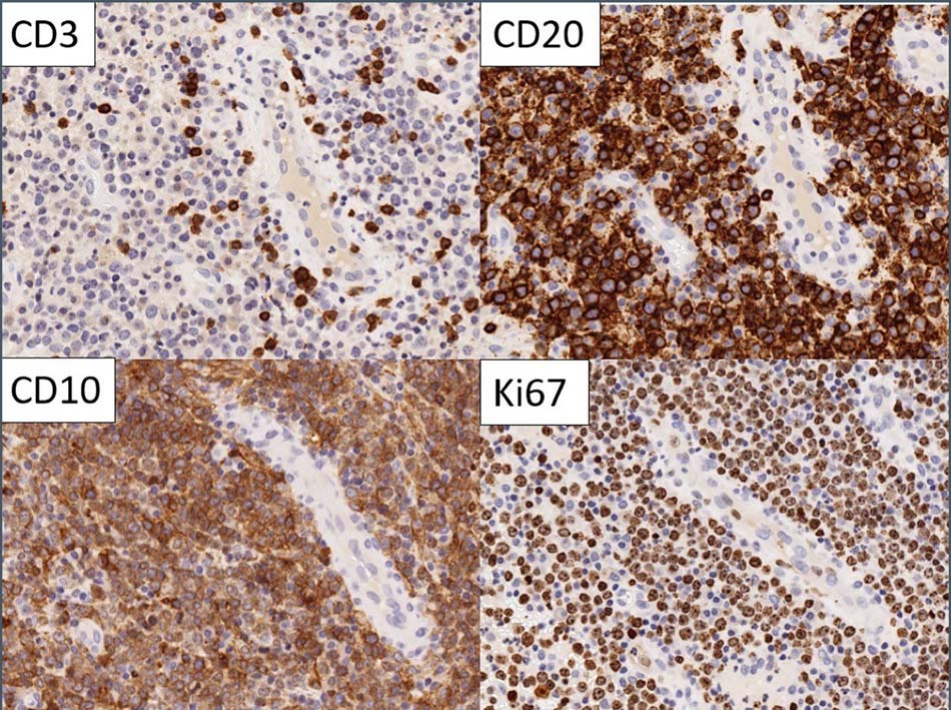
Immunohistochemistry: the large cells expressed CD20 and CD10 and showed high Ki67 proliferation rate. Small reactive T cells were present in the background (CD3).

A 42-year-old female was referred for the investigation of anal pain for which 4 weeks earlier she had undergone an incision and drainage of a perianal abscess at a teaching hospital. Pus for culture (but no tissue sample) was taken. On examination, there was an external opening evident in the 4 o’clock position about 2 cm from the anal verge. In the light of an altered bowel habit, she underwent colonoscopy with no additional findings. EUA confirmed a trans-sphincteric fistula involving the lower one-third of the external anal sphincter. The distal part of the tract was partially excised for histology and a loose seton inserted. Histopathology showed a sinus tract lined by inflammatory granulation tissue but which was infiltrated by sheets of lymphocytes and plasma cells with clusters of medium and large lymphocytes, centroblasts and immunoblasts. The cells showed no viral cytopathic effects. Adjacent mature fibrous tissue showed patchy mixed inflammatory cell infiltration (Figs 1 and 2). Immunohistochemical staining confirmed a diffuse large B cell lymphoma showing CD20, BCL6 and CD10 positivity but which was BCL-2 and MUM-1 negative. There was high (>90%) Ki67 staining and slides were EBV-negative (Fig. 3). Monoclonal IGH and IGK gene rearrangements were detected consistent with a diagnosis of B cell clonality. HIV status was negative.

**Figure 4:**
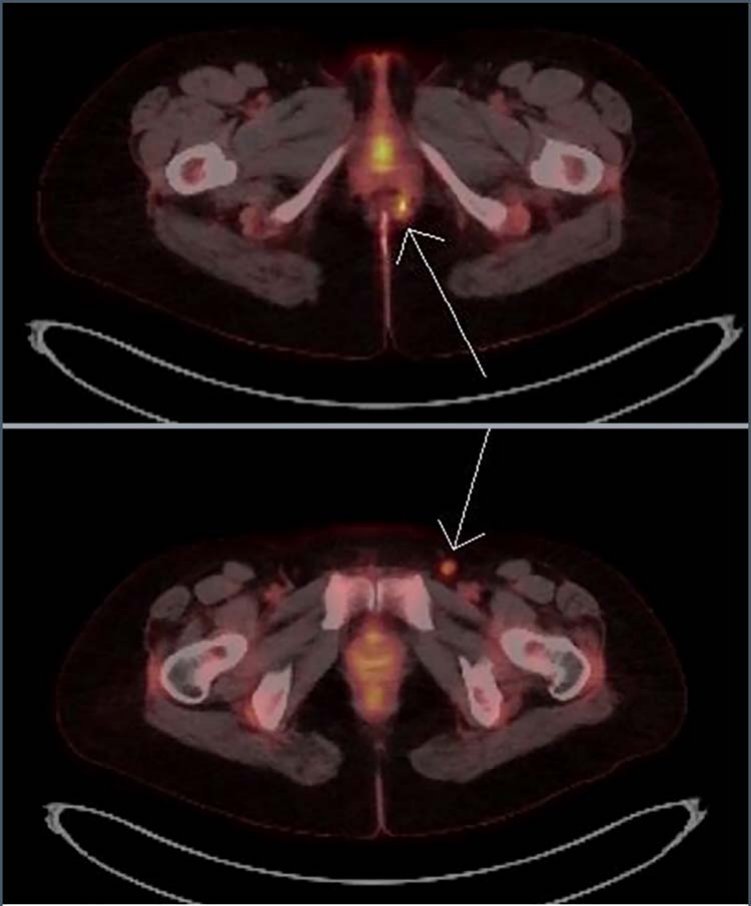
Transaxial PET scan. Upper view shows moderately intense uptake in the left perianal region. Lower view shows mild uptake in the left inguinal node.

Imaging with MRI showed a trans-sphincteric fistula crossing the junction of the middle and lower third of the anal sphincters with a prominent left inguinal lymph node. A PET scan (Fig. 4) showed a moderately intense uptake in the left perianal region around the fistula with bilateral inguinal uptake (more so on the left side). Biopsy of the left inguinal node showed no evidence of lymphoma.

**Figure 5:**
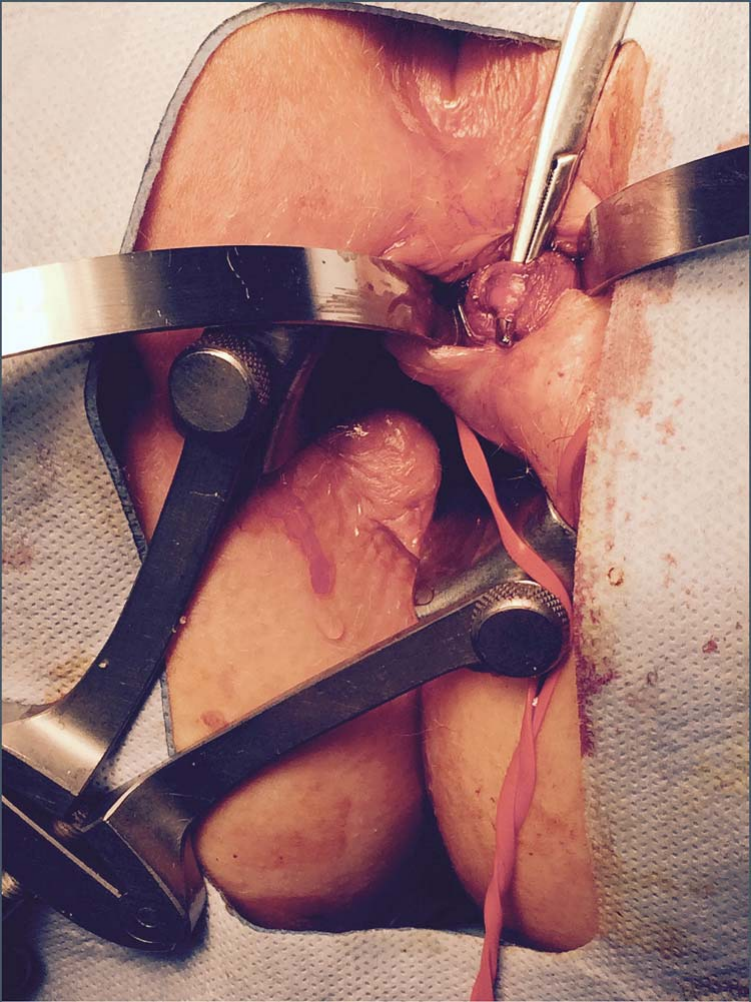
Operative photograph demonstrating the fistula tract

The patient was commenced on R-CHOP (Cyclophosphamide, Doxorubicin, Vincristine and Prednisone plus Rituxan), and after 3 cycles, there was a complete metabolic response on repeat PET scanning. Three months after surgery, the seton became dislodged. A repeat EUA was performed with reinsertion of the seton and an area of induration was noted between the 9 and 11 o’clock positions on the contralateral side, although without any evidence of an internal opening. The patient underwent a further 2 cycles of R-CHOP therapy. Repeat MRI confirmed the left-sided fistula with a new active tract on the right-hand side. This developed into a right perianal abscess which underwent incision and drainage. Biopsy of the right side showed no evidence of lymphoma. A left LIFT procedure was subsequently performed with ligation of the main tract in the inter-sphincteric space and a superficial secondary tract on the right side was divided and ligated. Histology showed no lymphoma. Infection in the inter-sphincteric space led to recurrence but repeat LIFT procedure plus a seton in the inter-sphincteric space resulted in closure (Fig. 5). The fistula has remained closed at 3 years of follow-up with no evidence on repeat MRI of active sepsis.

## DISCUSSION

Primary lymphoma of the anal canal represents only about 0.1% of all anal malignancies [9]. The majority presents as perianal sepsis in immunocompromised patients who most commonly have HIV-AIDS [6]. In these cases, the Epstein–Barr virus is frequently demonstrable by *in-situ* hybridization [8, 10].

Similarly to our case, Yaprak *et al.* [7] have reported that PET scanning in anal lymphoma shows pathological FDG uptake in the perineum. In localized disease, standard CHOP chemotherapy with the addition of Rituximab can be curative. Although localized lymphoma of the anal canal may necrose and fistulate, this would appear to be exceedingly rare. This raises the possibility conversely that a low-grade subclinical fistula could be supervened by lymphomatous change. In our case, in favour of the latter suggestion is the finding of sheets of lymphoma cells within the fistula rather than encasing the main tract. The first report of an anal B cell lymphoma by Steele *et al.* [5] proposed that tumour developing in an anal gland could obstruct its duct and lead to a fistula. From the course of this case, it would seem less likely that a lymphoma would preferentially grow within a fistula of its own making.

In perianal sepsis, the finding of a persistent pro-inflammatory cytokine profile in cryptoglandular fistulas [11] can precede malignant transformation where Wang *et al.* [12] have demonstrated polymorphisms in the expression of oxidative stress genes that are associated with the presence of NHL. Both of these latter findings would support the notion that there could be lymphomatous transformation within a pre-existing anal fistula rather than mere fistulation of a pre-existing anal lymphoma. This view, which suggests an alternative pathogenesis of rare anal cancers, might be supported by the finding that there have been unexpected single-point mutations in other tumours when they are located in the peri-anal region [13].

In summary, we have highlighted a rare case of B cell NHL of the anal region in an HIV-negative, non-immunocompromised patient. The course of this case raises the prospect that the lymphoma supervened on the fistula. In this patient, the lack of features in the initial presentation which suggested anything other than a simple fistula reinforces the importance of routine histopathology of perianal disease particularly where there is an underlying mass effect or in recurrent cases.
